# Microstructure and Properties of CNTs/2A12 Aluminum Matrix Composites Fabricated via Additive Friction Stir Deposition

**DOI:** 10.3390/ma19010112

**Published:** 2025-12-29

**Authors:** Zhiguo Lei, Mengran Zhou, Jiasheng Cao, Gaoqiang Chen, Shicheng Xu, Yu Xue, Yating Zhang, Qingyu Shi

**Affiliations:** 1 State Key Laboratory of Clean and Efficient Turbomachinery Power Equipment, Department of Mechanical Engineering, Tsinghua University, Beijing 100084, China; anderson9600lzy@163.com (Z.L.); 15922826867@163.com (J.C.); cheng1@tsinghua.edu.cn (G.C.); shqy@mail.tsinghua.edu.cn (Q.S.); 2 Key Laboratory for Advanced Materials Processing Technology, Ministry of Education, Beijing 100084, China; 3 Beijing Xinfeng Aerospace Equipment Co., Ltd., Beijing 100854, China; xsc1355788923@163.com (S.X.); xueyu1497@163.com (Y.X.); zhyt3521@163.com (Y.Z.)

**Keywords:** aluminum matrix composites, carbon nanotubes, Al-Cu-Mg alloy, additive friction stir deposition, microstructure, mechanical properties, corrosion resistance

## Abstract

Carbon nanotubes/2Al2 composites, due to their low density, high specific strength, and high elastic modulus, are representative lightweight structural materials for next-generation aerospace applications. Traditional processing methods are inefficient and have long production cycles, making them unsuitable for the demands of efficient, rapid, and intelligent manufacturing of complex structures. This article proposes the use of metal additive manufacturing technology to solve this problem. For the first time, a 22 mm high carbon nanotube/2Al2 composite was fabricated using additive friction stir deposition, and the changes in surface morphology, microstructure, mechanical properties, and corrosion resistance of the as-deposited composite were systematically studied. After additive manufacturing, the composite exhibited a continuous and defect-free, typical onion-like structure. The as-deposited microstructure consists of uniformly equiaxed grains with an average grain size of 1.23 μm to 1.62 μm and uniformly distributed Al_2_Cu particles. The tensile strength and elongation of the as-deposited composite in both the transverse and processing directions are no less than 450 MPa and 15%, respectively, superior to those of the base material. After additive manufacturing, the as-deposited composite exhibited a corrosion current density of 0.19 μA cm^−2^ in the transverse direction—only 4% of that of the base material. This enhanced corrosion resistance is attributed to the uniform distribution of precipitated phases achieved through additive manufacturing, which suppresses micro-galvanic corrosion, resulting in minimal, uniform corrosion. This study provides a research foundation and technical support for the additive manufacturing of aluminum-based composites.

## 1. Introduction

In recent years, with the increasing severity of global climate change and the promotion of the “dual carbon” strategic goal, the aerospace industry’s increasing demand for lightweighting of complex-shaped structural components has led to the adoption of carbon nanotube/Al–Cu–Mg composites as a solution, owing to their low density, high elastic modulus, high specific strength, and excellent corrosion resistance [1,2]. CNTs are used as reinforcing particles to significantly improve the comprehensive performance of aluminum matrix composites through a matrix-reinforcement synergistic mechanism [3,4,5]. Although AMCs have made progress driven by performance, their preparation process still faces major technical bottlenecks. Traditional methods for manufacturing CNTs/Al composite parts, such as casting, often have two problems. On the one hand, high residual stress and porosity defects require a lot of post-processing [6]; on the other hand, traditional processing methods have technical pain points, including many processing steps, long production cycles, and difficulties with the integrated processing of complex-shaped structural parts [6]. These limitations make it difficult to meet the demands of rapid and intelligent manufacturing of complex-shaped AMC structural parts in aerospace and other fields. Additive manufacturing technology meets the above requirements from a technical principle perspective and is regarded as the preferred solution, which has brought about a revolutionary change in the manufacturing of complex geometries, providing high material efficiency and reducing waste [7,8].

Mainstream additive manufacturing (AM) technologies, including selective laser melting (SLM) and other melting-based AM techniques, face fundamental limitations in their process principles, hindering their large-scale application for aluminum matrix composites (AMCs) [9,10]. Aluminum alloys exhibit high laser reflectivity and low energy absorption, resulting in inefficient material melting and reduced processing efficiency [11]. Hakami et al. [12] demonstrated that melting-based AM methods inevitably encounter densification challenges during AMC preparation. Additionally, SLM and electron beam melting (EBM) are unsuitable for aluminum alloys due to their high thermal conductivity and propensity for hot cracking [13]. Additive friction stir deposition (AFSD) technology, based on friction stir welding (FSW) principles, fundamentally avoids the technical issues of densification and metallurgical defects associated with melting processes. As a solid-state joining technique, FSW is renowned for producing dense joints with superior mechanical properties [14,15]. This process minimizes common metallurgical defects such as porosity and cracks while promoting fine-grained, uniform microstructures [16,17]. AFSD represents a solid-state AM technology that synergizes the solid-state forming principles of FSW with the design flexibility of AM [18,19]. During solid-state forming, frictional heat generated between the feedstock and substrate induces local thermal softening and plastic flow of the material [20,21,22], enabling controlled deposition. As a thermostatically controlled process, solid-state AM triggers dynamic recrystallization mechanisms [23,24,25], facilitating the formation of equiaxed grains and thereby enhancing mechanical properties. Recent literature highlights successful applications of AFSD for AMC preparation [26,27,28]. Zhang et al. [29,30,31] achieved dense TiC/Al-Cu-Mg composites with uniform mechanical properties using AFSD, while Feng et al. [32] demonstrated single-pass multi-layer printing capabilities and revealed hardness variations along the building direction (BD) due to thermal cycling. Hahn et al. [33] reported mechanical properties in AA7050 aluminum alloys fabricated via AFSD that approached those of forged samples. These findings collectively validate the technological potential of AFSD for metal matrix composite fabrication. Notably, AFSD-processed 2195 Al-Li alloys exhibit significant performance anisotropy along the BD, with the top layer demonstrating superior mechanical properties (yield strength: 253.7 MPa, tensile strength: 409.4 MPa, elongation: 25.6%) compared to lower layers [34]. This layer also achieved peak tensile and yield strengths, with a corresponding hardness distribution trend [35]. The enhanced performance of the top layer is attributed to minimal thermal cycling during AFSD, whereas accumulated heat dynamically heat-treated previously deposited layers, promoting precipitation strengthening phase growth and grain coarsening [36,37]. Sun et al. [38] successfully fabricated TiB_2_/7055 composites via AFSD, achieving excellent mechanical properties while investigating their microstructural evolution and mechanical behavior.

However, no published literature exists on CNTs/2A12 composites manufactured through AFSD. This study successfully fabricated single-pass multi-layer CNTs/2A12 composites using AFSD, employing multi-scale characterization techniques to elucidate microstructure–property relationships. The findings provide a theoretical foundation and process guidance for the solid-state additive manufacturing of aluminum matrix composites.

## 2. Materials and Methods

**Figure 1:**
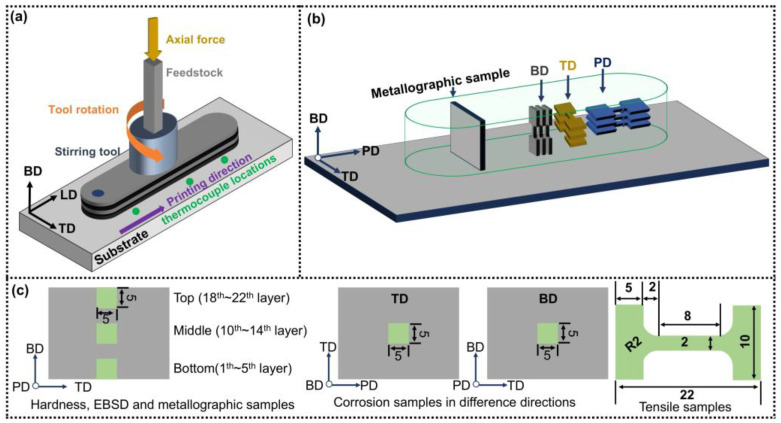
(**a**) Schematic diagram of AFSD process; (**b**) sampling position for microstructure analysis and tensile test; (**c**) sample dimensions.

In this study, the base material (BM) consisted of commercially available extruded CNTs/2A12 composite rods, which were subsequently processed into feedstocks with cross-sectional dimensions of 10 mm × 10 mm, a length of 400 mm, and a substrate thickness of 7 mm. The chemical composition of the BM is presented in Table 1. AFSD processing was conducted using a solid-state friction stir apparatus (Beijing Super Synchronous Co., Ltd., Beijing, China) developed by Tsinghua University. A schematic illustration of the AFSD process configuration is provided in Figure 1a. Under optimized process parameters (800 r min^−1^ rotational speed and 30 mm min^−1^ transverse velocity), 22-layer as-deposited samples were successfully fabricated, each with a layer thickness of 1 mm, a length of 150 mm, and a width of 29 mm. Temperature monitoring during processing was performed using three K-type thermocouples embedded within the BM substrate, with their specific locations schematically illustrated in Figure 1a. For microstructure characterization and mechanical performance testing, samples from both the as-deposited composite and the BM were extracted using wire electrical discharge machining (WEDM), with detailed sampling instructions provided in Figure 1b,c.

The surface onion-like structure of the as-deposited sample was analyzed using confocal laser scanning microscopy (CLSM; Zeiss LSM 900, Oberkochen, Germany). Metallographic samples were ground and mechanically polished, then etched with Keller’s reagent (Beijing Xinding Pengfei Technology Development Co., Ltd., Beijing, China) for 3–6 s. The macro-morphology of the cross-section along the build direction (BD) of the as-deposited sample was observed by optical microscopy (OM; Olympus GX71, Tokyo, Japan), while the microstructure of the BM and as-deposited sample was observed by scanning electron microscopy (SEM; ZEISS Gemini 300, Oberkochen, Germany) at an acceleration voltage of 5 kV. The elemental distribution of the BM and the as-deposited sample was analyzed using energy-dispersive X-ray spectroscopy (EDS; Oxford Aztec X-Max50, Billerica, MA, USA) at an acceleration voltage of 15 kV. Electron backscatter diffraction (EBSD; Oxford Aztec HKL Symmetry S2, Billerica, MA, USA) is used to observe the grain size and high-angle grain boundaries of the BM and as-deposited sample. The samples were prepared by electropolishing, which was carried out on the equipment (EP-3000, Beijing Tongde entrepreneurship technology Co., Ltd., Beijing, China) with a polishing parameter of 10 vol% perchloric acid alcohol solution (Cangzhou Xinyuanquan Chemical Co., Ltd., Tianjin, China) at 20 V, 25 °C, for 4–10 s. The phase composition was analyzed by XRD (XRD; Bruker D8, Bruker, Billerica, MA, USA) at that parameter of 40 kV and 30 mA, and scanning angles from 20° to 80°, Cu Ka, 2° min^−1^. The structural damage of carbon nanotubes of the BM and as-deposited sample was analyzed by Raman spectroscopy (Alpha 300 R with TEM_00_ laser, WITec, Ulm, Germany), using a parameter of the testing scope that ranged from 1000 cm^−1^ to 2000 cm^−1^. Microhardness testing was conducted on a microhardness tester (Future-tech FM-810, Saitama, Japan), using a 300 gf load with a holding time of 10 s. Tensile testing was performed on a universal testing machine (SHIMADZU, MTS E45, Eden Prairie, MN, USA) at a strain rate of 3.3 × 10^−4^ s^−1^. Dog-bone tensile samples had a thickness of 2 mm and a gauge length of 8 mm. Electrochemical impedance spectroscopy (EIS) and potentiodynamic polarization measurements were carried out via a Zahner electrochemical workstation (Zahner Elektrochemie Zennium X, Kronach, Germany) with a standard three-electrode cell, utilizing a Pt counter electrode and Ag/AgCl reference electrode (saturated KCl). The test solution was a 3.5 wt.% NaCl solution (Jinan Shiji Tongda Chemical Co., Ltd., Jinan, China). EIS tests applied a 5 mV amplitude sinusoidal potential at frequencies ranging from 100 kHz to 10 mHz. Potentiodynamic polarization scans were performed from −1.8 V to −0.5 V at 1 mV/s. A 7-day immersion test was conducted in the 3.5 wt.% NaCl solution. Post-immersion corrosion morphology was analyzed by SEM.

## 3. Results and Discussion

### 3.1. Surface Morphology

**Figure 2:**
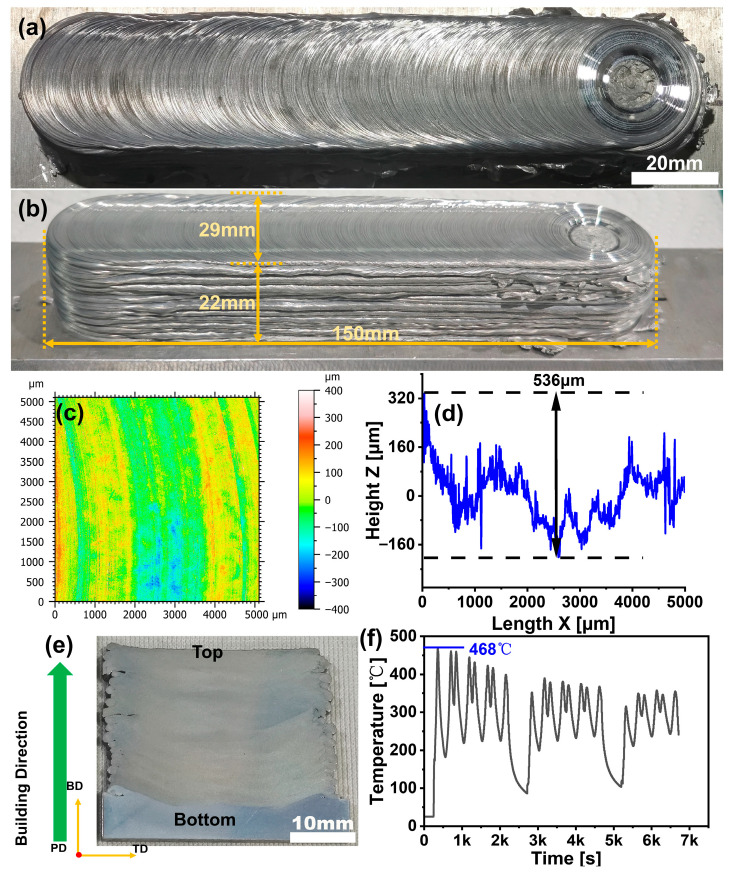
Macrograph image and temperature monitoring data of as-deposited composite: (**a**) onion-ring surface morphology; (**b**) side view; (**c**) CLSM image of onion-ring structure; (**d**) surface height variation; (**e**) cross-sectional view; (**f**) temperature profile.

For the first time, a CNTs/2A12 composite with dimensions of 150 mm (L) × 29 mm (W) × 22 mm (H) was successfully fabricated using AFSD. The surface morphology of the as-deposited composite is presented in Figure 2. As shown in Figure 2a, the surface of the as-deposited composite exhibits characteristic onion-ring structures [38] under the processing conditions of 800 r min^−1^ and 30 mm min^−1^. Figure 2b demonstrates the side-view morphology, revealing excellent layer-by-layer printing quality, a smooth surface profile, and no observable structural collapse. Confocal laser scanning microscopy analysis (Figure 2c) confirms that the as-deposited sample surface maintains uniformity and continuity, being entirely free of cracks or defects, with a measured height difference of 536 μm (Figure 2d). The metallographic image of the cross-sectional sample (Figure 2e) further indicates that the as-deposited material exhibits homogeneous and dense microstructure without any visible voids or cracks. The absence of observable defects can be attributed to the optimized processing parameters. Specifically, (1) the AFSD process operates at a maximum temperature of 468 °C (Figure 2f), which remains below the melting point of aluminum, thereby preventing void formation caused by material remelting and rapid cooling; (2) the feedstock undergoes complete plastic flow under combined extrusion and shearing forces, ensuring effective material deposition [39,40,41,42].

### 3.2. Microstructure

**Figure 3:**
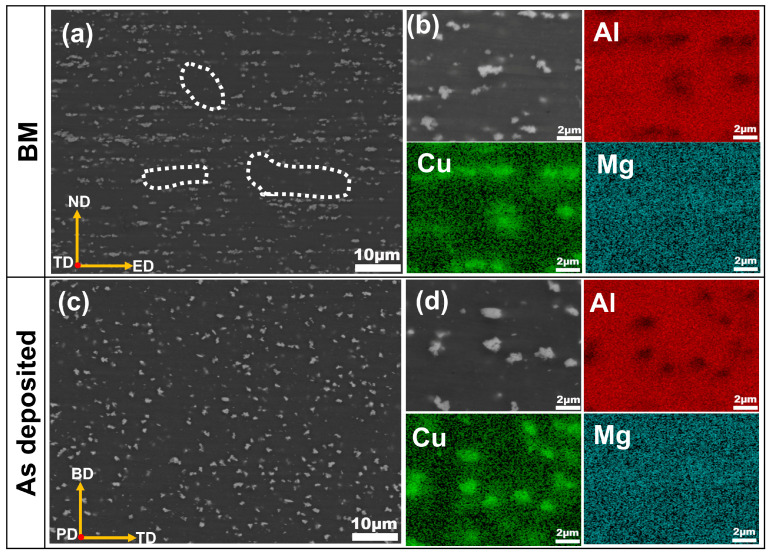
Backscattered electron (BSE) images and energy-dispersive X-ray spectroscopy (EDS) elemental mappings of (**a**,**b**) BM and (**c**,**d**) as-deposited composite.

Figure 3 presents backscattered electron (BSE) images and energy-dispersive X-ray spectroscopy elemental mappings for both the BM and the as-deposited sample. As shown in Figure 3a,c, the precipitated phases in the BM exhibit agglomeration and preferential alignment along the extrusion direction. In contrast, the as-deposited composite demonstrates a more uniform distribution of precipitated phases, which is attributed to the thermomechanical coupling effects during AFSD processing [42]. EDS analysis (Figure 3b,d) confirms that the precipitated phases in both the BM and as-deposited composite are primarily enriched in Al and Cu.

**Figure 4:**
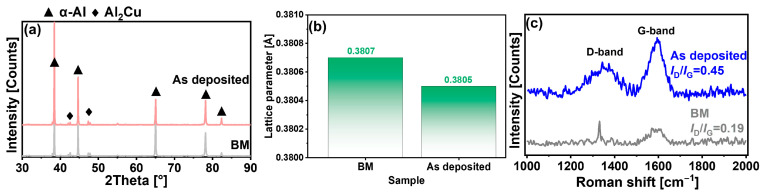
(**a**) XRD patterns of the BM and as-deposited composite; (**b**) α-Al lattice parameters of the BM and as-deposited composite; (**c**) Raman spectra of the BM and as-deposited composite.

To further investigate the phase transformation characteristics, X-ray diffraction analysis was conducted on both the BM and as-deposited composite. Figure 4a reveals nearly identical XRD patterns for both materials, dominated by α-Al and Al_2_Cu phases. However, quantitative analysis shows a reduction in the diffraction peak intensity of Al_2_Cu and a decrease in the lattice parameter of α-Al (Figure 4b). This phenomenon can be attributed to the heating of the composite to 468 °C during AFSD processing, which approximates the solution treatment temperature for Al-Cu-Mg alloys. Given that the atomic radius of copper is smaller than that of aluminum, this thermal treatment results in a reduction in the aluminum lattice constant [43,44].

Raman spectroscopy was employed to assess the structural integrity of carbon nanotubes (CNTs) in the composite (Figure 4c). Characteristic vibrational bands were observed at approximately 1350 cm^−1^ (D-band, associated with structural defects) and 1590 cm^−1^ (G-band, indicative of graphitic ordering) [45]. The intensity ratio of the D-band to G-band (I_D/I_G) serves as a quantitative measure of CNTs’ structural damage. After AFSD processing, the I_D/I_G ratio in the CNTs/2A12 composite increased from 0.19 in the base material (BM) to 0.45, indicating that the structural damage incurred during AFSD processing remained within acceptable limits [46].

It should be noted that compromised CNTs’ integrity and the presence of defective carbon nanotubes can significantly impair their load transfer capability [47,48]. Compared to conventional methods such as high-energy ball milling, which often introduce substantial defects and strain, potentially leading to structural defects or reduced crystallinity of carbon nanotubes [48], AFSD demonstrates superior performance in minimizing damage to CNTs. By avoiding these harsh processing conditions, AFSD facilitates the production of higher-quality CNTs with fewer defects.

**Figure 5:**
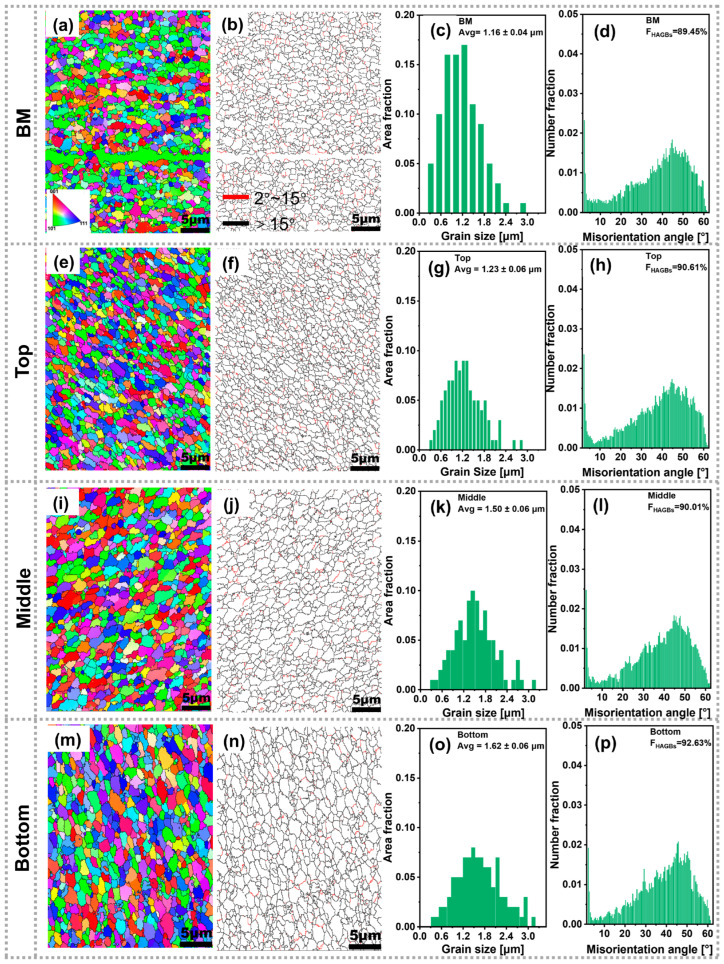
Inverse pole figure (IPF) maps and corresponding grain/grain boundary data: (**a**–**d**) BM; (**e**–**h**) top; (**i**–**l**) middle; (**m**–**p**) bottom.

To investigate the microstructural evolution of the BM and the as-deposited composite, electron backscatter diffraction was employed to characterize the grain size and high-angle grain boundaries (HAGBs) of both materials across the top, middle, and bottom regions of the cross-section. Figure 5 displays inverse pole figure (IPF) maps, grain boundary (GB) maps, average grain size distribution maps, and misorientation angle distribution maps for the BM and the as-deposited composite. In the GB maps, red lines represent low-angle grain boundaries (LAGBs), defined by misorientation angles ranging from 2° to 15°, whereas black lines denote HAGBs, with misorientation angles exceeding 15°. As shown in Figure 5a, the extruded BM exhibits elongated and non-uniform grains, indicating a random grain orientation distribution.

In contrast, after AFSD, the grain morphology of the as-deposited composite transitions into uniformly equiaxed grains across all cross-sectional regions (Figure 5e,i,m). This transformation is attributed to dynamic recrystallization occurring within each deposited layer during AFSD [24,25]. Grain size statistics (Figure 5c,g,k,o) reveal average grain sizes of 1.23 μm, 1.50 μm, and 1.62 μm for the top, middle, and bottom regions of the as-deposited composite, respectively. Notably, the average grain size in all regions of the as-deposited composite slightly exceeds that of the BM (1.16 μm), with the bottom region exhibiting the coarsest grains. Furthermore, the bottom region also contains the highest number of HAGBs (Figure 5p). This modest grain growth is likely driven by repetitive thermal cycles and severe plastic deformation during deposition [49]. The enhanced grain coarsening in the bottom region suggests that it experiences more frequent thermal cycling compared to other regions [7,15]. In summary, both the BM and the as-deposited composite exhibit average grain sizes within the range of 1–2 μm, indicating excellent thermal stability of the as-deposited material.

### 3.3. Mechanical Properties

**Figure 6:**
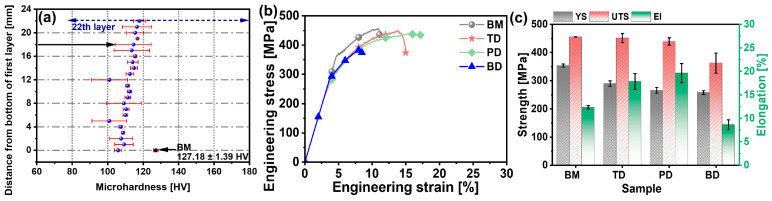
Mechanical properties of BM and as-deposited composite along different directions: (**a**) microhardness; (**b**) engineering strain–stress curves; (**c**) UTS, YS, and elongation in the as-deposited composite and BM.

Figure 6a presents the hardness distribution along the cross-sectional BD of the as-deposited composite and the BM. The as-deposited composite exhibits peak hardness at the 22nd layer, reaching approximately 94% of the BM’s hardness value. Below the 16th layer, the hardness progressively decreases, reaching its minimum at the substrate/first-layer interface. During deposition, the consumable rod undergoes dynamic recrystallization, transforming its fibrous grain structure into an equiaxed morphology with moderate coarsening tendencies. As a heat-treatable precipitation-strengthened alloy, 2A12 primarily derives its strengthening from secondary phases, which play a critical role in hardness enhancement. XRD analysis (Figure 4b) confirms partial dissolution of these precipitates into the matrix. This dissolution reduces their effectiveness in pinning dislocations, thereby contributing to the observed hardness reduction.

The tensile properties of the base material (BM) and the as-deposited composite along the TD, PD, and BD were evaluated. The collected data are presented as engineering stress–strain curves and bar charts in Figure 6b,c. During tensile testing, the BM exhibits significant work hardening (Figure 6b), which is attributed to increased dislocation multiplication and interactions during plastic deformation. This phenomenon enhances resistance to deformation, thereby increasing strength and hardness. The Al_2_Cu phases in the Al-Cu-Mg alloy further impede dislocation motion, amplifying the work-hardening effect. A similar work-hardening behavior was observed in the as-deposited composite along the TD and PD, whereas the BD exhibited negligible work hardening. Figure 6c summarizes the average tensile properties, revealing comparable yield strength (289 MPa), ultimate tensile strength (UTS, 451 MPa), and elongation (16%) between the TD and PD. In contrast, the BD sample showed significantly reduced tensile strength and elongation (with ~8% elongation—approximately half that of the TD). This degradation stems from weaker interlayer bonding compared to intralayer cohesion. Relative to the extruded BM (UTS: 455 MPa, YS: 354 MPa, El: 12%), the TD and BD samples exhibited marginally lower strength but superior elongation. Overall, the tensile properties of the as-deposited composite remained competitive with those of the BM. This balance arises because age hardening constitutes the primary strengthening mechanism in heat-treatable 2A12 alloy. While repeated thermal cycles and plastic deformation during asymmetric friction stir deposition (AFSD) promoted grain coarsening and precipitate dissolution, the concurrently elevated dislocation density partially compensated for this strength deficit.

**Figure 7:**
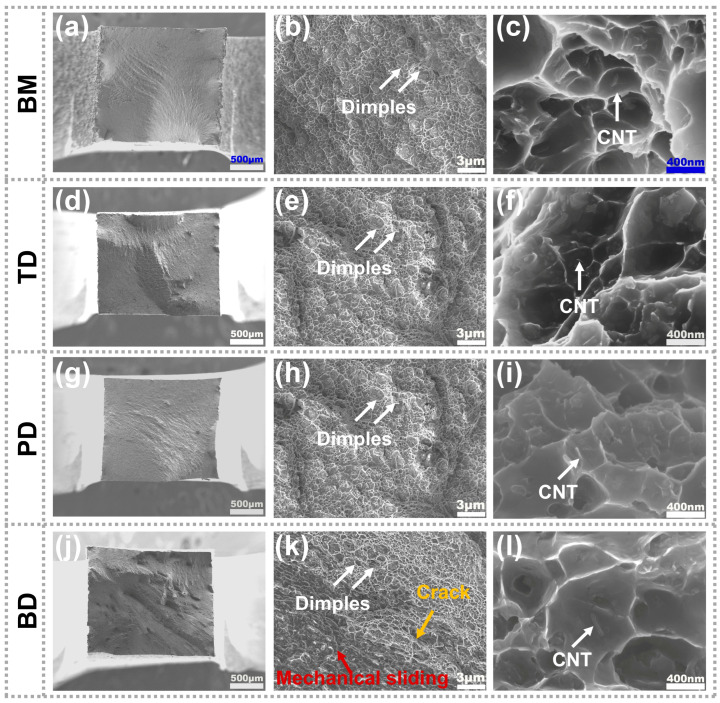
Tensile fracture surfaces of the BM and as-deposited composite along different directions: (**a**–**c**) BM; (**d**–**f**) top; (**g**–**i**) middle; (**j**–**l**) bottom.

Figure 7 presents the tensile fracture surfaces of the BM and as-deposited composite along the TD, PD, and BD. Figure 7a reveals fibrous zones across the BM fracture surface at low magnification. Figure 7b displays uniformly distributed dimples of varying sizes, indicative of ductile fracture, with fragmented secondary-phase particles observable within larger dimples. Figure 7c shows that carbon nanotubes (CNTs) at the bottom of the dimples play a role in load transmission [50]. The TD and PD samples exhibited characteristic ductile fracture morphology (Figure 7d,i). In contrast, the BD fracture surface showed localized zones featuring mechanical sliding and cleavage patterns—characteristics indicative of mechanically interlocked fracture (Figure 7k). These observations demonstrate that insufficient thermomechanical conditions during deposition prevented complete metallurgical bonding at interfaces exhibiting mechanical sliding features [51].

### 3.4. Corrosion Resistance

**Figure 8:**
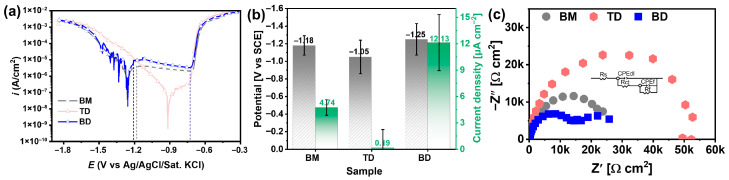
The electrochemical performance of the BM and as-deposited composite along different directions: (**a**) potentiodynamic polarization curves; (**b**) quantitative corrosion parameters derived from polarization curves; and (**c**) Nyquist curves.

Figure 8 presents the potentiodynamic polarization curves of the BM and the as-deposited composite along the TD and BD after 60 min of immersion in a 3.5 wt.% NaCl solution. As shown in Figure 8a, the BM exhibits a distinct passivation window within the potential range of −1.21 V to −0.78 V, where the matrix dissolves at an extremely low current density. This behavior is attributed to the formation of dense and stable corrosion products, which effectively prevent corrosive media from contacting the matrix surface. A sudden current surge occurs on the anodic polarization curve as the applied potential increases, indicating the breakdown of the surface film formed during corrosion. However, this film provides minimal protection, leading to rapid dissolution of the aluminum alloy. The as-deposited composite along both BD and TD directions also exhibits a passivation window. Notably, the BD sample demonstrates a wider passivation window compared to the BM, while the TD sample displays a pseudo-passivation region. This pseudo-passivation behavior is attributed to more uniform corrosion in the NaCl solution, with minimal corrosion product formation. The corresponding corrosion potential (Ecorr) and corrosion current density (Icorr) derived from the polarization curves are summarized in Figure 8b. The TD sample exhibits the lowest Icorr (0.19 μA cm^−2^), representing only 4% of the BM’s value. In contrast, the BD sample shows the highest Icorr, reaching 255% of the BM’s value. Electrochemical impedance spectroscopy (EIS) measurements were also conducted, with results shown in Figure 8c. All three Nyquist curves exhibit a semi-circular characteristic. Among them, the TD sample has the largest semi-circular diameter, indicating the best corrosion resistance, which is consistent with the polarization curve results.

Figure 8c presents the EIS curves of the BM and as-deposited composite samples after 30 min of immersion in a 3.5 wt.% NaCl solution. The equivalent circuit (EC) employed for fitting the EIS data is illustrated in Figure 8b, with the corresponding fitting parameters summarized in Table 2. In the EC model, R_s_ represents the solution resistance, R_f_ denotes the resistance of the surface oxide film, and R_ct_ correlates with the charge transfer process at the interface between the aluminum substrate and the electrolyte. As shown in Table 2, the as-deposited composite sample in the TD exhibits the lowest Rf value, suggesting the absence of dense corrosion product formation. This observation aligns with the polarization curve results. Notably, R_ct_ serves as a critical parameter for evaluating corrosion resistance. The TD sample demonstrates a significantly higher R_ct_ value (13,470 Ω cm^2^) compared to other samples, indicating effective inhibition of charge transfer kinetics at the metal–electrolyte interface.

Based on the aforementioned results, when the BM and as-deposited composite samples are exposed to a corrosive environment, the oxide film exhibits fragility and undergoes rapid breakdown under chloride ion erosion, leading to the formation of chloride ion channels. These are particularly pronounced in the as-deposited composite sample along the TD. Upon contact with the corrosive solution, corrosion products form on the sample surface. A higher R_ct_ value indicates an elevated energy barrier for electron transfer from the metal matrix to the corrosive medium, thereby significantly impeding electron migration and inhibiting electrochemical reactions. The corrosion products restrict the corrosion behavior of the as-deposited composite in the TD, effectively reducing the corrosion rate. This demonstrates that the TD sample exhibits superior corrosion resistance compared to other configurations.

**Figure 9:**
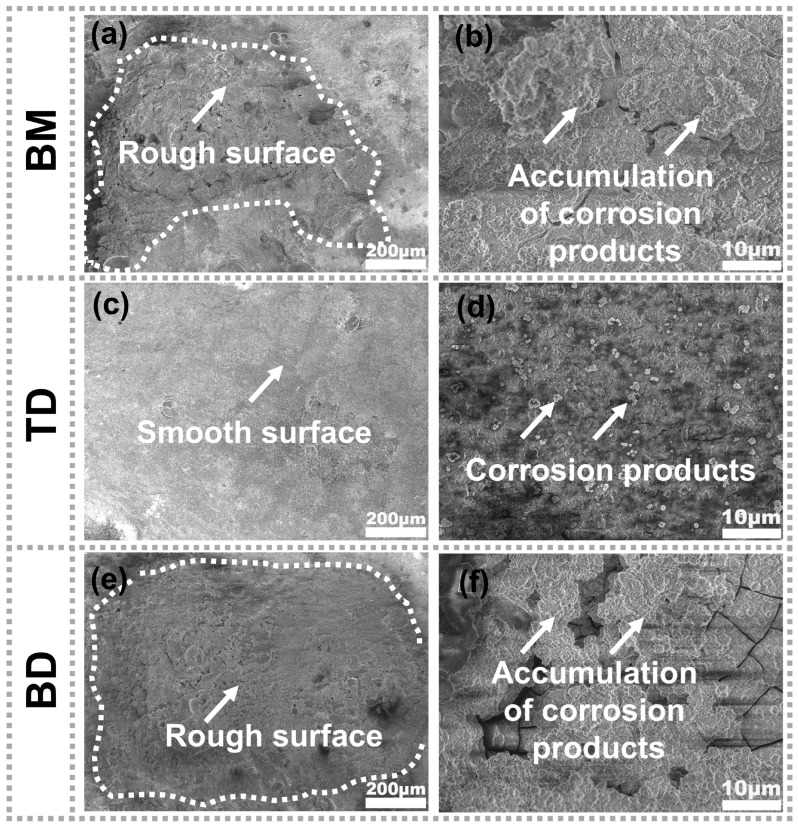
SEM micrographs of the BM and the as-deposited composite along different directions after immersion testing: (**a**,**b**) BM; (**c**,**d**) TD; (**e**,**f**) BD.

Figure 9 presents the typical corrosion surface morphology of the BM and as-deposited samples after 7 days of immersion in a 3.5 wt.% NaCl solution. As shown in Figure 9a, the BM surface is severely damaged after immersion, with numerous loose corrosion products forming on its rough surface. This observation aligns with the polarization curve results (Figure 8a), where the non-protective nature of the BM surface film facilitates rapid aluminum alloy dissolution and localized corrosion. In contrast, the corrosion surfaces of the as-deposited composites remain relatively smooth, devoid of large corrosion pits. Figure 9b reveals that the TD sample maintains a smooth surface throughout immersion, with uniform corrosion occurring and only minimal corrosion product formation. Conversely, the sample along the BD exhibits more severe corrosion than the BM (Figure 9a,e), characterized by extensive corrosion product accumulation—a finding consistent with the polarization curve data. In corrosive environments, the influence of grain size on alloy corrosion behavior follows the Hall-Petch relationship, where grain refinement promotes protective oxide film formation and enhances corrosion resistance [52,53]. Grain boundaries exhibit higher energy states and chemical activity than grain interiors, facilitating passive film nucleation [54]. As shown in Figure 5, the BM exhibits a smaller average grain size (1.16 μm) than the as-deposited composite. Both materials contain abundant grain boundaries, with higher boundary density in the BM. This microstructure explains the distinct passivation behavior observed in the polarization curves (Figure 8a), including the BM’s extended passive plateau and elevated pitting potential.

However, precipitation-strengthened alloys introduce secondary phases that form micro-galvanic couples with the matrix due to potential differences. These couples accelerate localized corrosion through preferential dissolution of the anodic constituent [55,56,57,58]. In the 2A12 alloy, Al_2_Cu precipitates exhibit an electrode potential of −0.70 V vs. SCE, contrasting with the α-Al matrix (−0.85 V vs. SCE) [59]. In the extruded BM, agglomerated Al_2_Cu precipitates act as cathodic sites, exacerbating matrix dissolution. Following additive processing, the sample along the TD demonstrates partial dissolution and homogeneous dispersion of precipitates, thereby reducing susceptibility to micro-galvanic corrosion—a phenomenon consistent with its ultralow corrosion current density (0.19 μA·cm^−2^, Figure 8b). In contrast, the sample along the BD exhibits the highest corrosion current density (255% of the BM value). This is attributed to inferior interlayer bonding, where interfacial gaps increase the active surface area exposed to Cl^−^ ingress, thereby accelerating corrosion kinetics.

### 3.5. Applications and Limitations

This study successfully demonstrates the additive manufacturing and enhanced corrosion properties of CNTs/2A12 composites fabricated using AFSD technology, offering technological innovation for the rapid intelligent manufacturing of aerospace composite structural components such as aircraft wings. Nevertheless, certain aspects warrant further improvement and investigation. Firstly, AFSD predominantly affects microstructural evolution during composite processing, rendering it difficult to achieve optimal microstructure throughout the entire bulk material. Secondly, although AFSD enables the fabrication of large-sized components via layer-by-layer deposition, the thermal stability of the as-deposited structures requires more comprehensive assessment. Moreover, the current strength of CNTs/2A12 composites remains insufficient for engineering applications, necessitating subsequent solution and aging heat treatments. Furthermore, the mechanism through which CNTs inhibit recrystallized grain growth remains unclear and demands further elucidation. Future research should employ more precise characterization techniques to better understand the role carbon nanotubes play in the recrystallization process.

## 4. Conclusions

This study successfully fabricated a 22-layer single-pass CNTs/2A12 composite via AFSD for the first time. Microstructural characterization and performance testing were employed to investigate changes in the microstructure and properties of the BM following the additive manufacturing process. The conclusions are as follows:

(1) The as-deposited composite exhibited no significant macro-defects, demonstrating that defect-free layer-by-layer deposition can be achieved under the employed process parameters at 800 rpm, 30 mm min^−1^.
(2) Under thermomechanical coupling effects, the as-deposited composite exhibited an average grain size ranging from 1.23 μm to 1.62 μm along the BD, indicating that grain size is unaffected by layer-by-layer thermal cycling.
(3) The tensile strength and elongation of the as-deposited composite in the TD and PD were comparable to the BM. However, the mechanical properties were lower in the BD than in the TD and PD due to inferior interlayer bonding.
(4) The as-deposited composite demonstrated significantly enhanced corrosion resistance in the TD, exhibiting only 4% of the BM’s corrosion rate. This improvement is attributed to precipitate dissolution, which mitigates galvanic corrosion and promotes more uniform corrosion.

## Figures and Tables

**Table 1:** Chemical composition of BM (wt.%).

Cu	Mg	Mn	Fe	Si	Zn	Ti	CNTs	Al
4.05	0.95	0.61	0.29	0.11	0.02	0.11	1.5	Balance

**Table 2:** Curve-fitted electrochemical parameters from the EIS spectra.

Samples	R_S_ (Ω·cm^2^)	Q_f_ (10^−5^F/cm^2^·s^−n^)	n_f_	R_f_ (Ω·cm^2^)	Q_dl_ (10^−5^F/cm^2^·s^−n^)	n_dl_	R_ct_ (Ω·cm^2^)	R_L_ (Ω·cm^2^)	L	R_f_ + R_ct_ (Ω·cm^2^)
BD	9.892	3.239	0.9113	4058	86.23	1	2593	/	/	6651
BM	8.996	2.105	0.8637	9151	38.78	1	2395	/	/	11,546
TD	7.652	0.417	0.9305	92.38	0.707	0.9039	13,470	4319	9259	13,562

## Data Availability

The raw data supporting the conclusions of this article will be made available by the authors on request.
